# (*E*)-*N*′-(4-Chloro­benzyl­idene)-*p*-toluene­sulfonohydrazide 0.15-hydrate

**DOI:** 10.1107/S1600536809014512

**Published:** 2009-04-25

**Authors:** Reza Kia, Hoong-Kun Fun, Hadi Kargar

**Affiliations:** a X-ray Crystallography Unit, School of Physics, Universiti Sains Malaysia, 11800 USM, Penang, Malaysia; b Department of Chemistry, School of Science, Payame Noor University (PNU), Ardakan, Yazd, Iran

## Abstract

The asymmetric unit of the title compound, C_14_H_13_ClN_2_O_2_S·0.15H_2_O, a novel sulfonamide derivative, comprises two crystallographically independent mol­ecules (*A* and *B*) and a water mol­ecule of crystallization, which is partially occupied. One of the mol­ecules (*B*) is disordered over two positions (*B* and *C*) with refined site occupancies of 0.605 (10) and 0.395 (10). The dihedral angles between the two benzene rings in mol­ecules *A*, *B* and *C* are 67.8 (3), 74.6 (5) and 84.96 (11)°, respectively. In the crystal structure, inter­molecular N—H⋯O and C—H⋯O hydrogen bonds link the components of the asymmetric unit. The crystal structure is further stabilized by inter­molecular π–π inter­actions [centroid–centroid distances = 3.4518 (10)–3.5859 (10) Å].

## Related literature

For bond-length data, see: Allen *et al.* (1987 ▶). For hydrogen-bond motifs, see: Bernstein *et al.* (1995 ▶). For related structures and applications, see, for example: Kia *et al.* (2008*a* ▶,*b* ▶); Mehrabi *et al.* (2008 ▶); Tabatabaee *et al.* (2007 ▶); Ali *et al.* (2007 ▶); Tierney *et al.* (2006 ▶); Krygowski *et al.* (1998 ▶). For the stability of the temperature controller used for the data collection, see: Cosier & Glazer (1986 ▶). For related literature on bioactivity, see: Kayser *et al.* (2004 ▶).
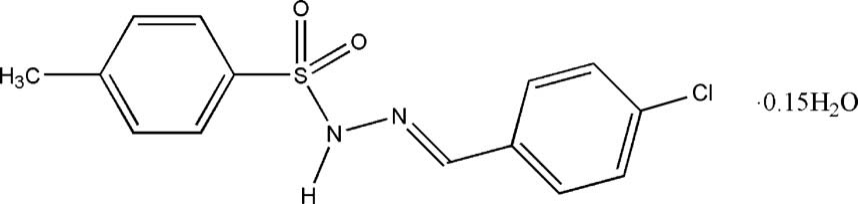


## Experimental

### 

#### Crystal data

C_14_H_13_ClN_2_O_2_S·0.15H_2_O
*M* *_r_* = 311.61
Triclinic, 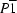

*a* = 7.9408 (2) Å
*b* = 11.0592 (2) Å
*c* = 17.7759 (4) Å
α = 77.521 (1)°
β = 83.415 (1)°
γ = 70.313 (1)°
*V* = 1433.60 (5) Å^3^
*Z* = 4
Mo *K*α radiation
μ = 0.42 mm^−1^
*T* = 100 K
0.58 × 0.13 × 0.05 mm

#### Data collection

Bruker SMART APEXII CCD area-detector diffractometer
Absorption correction: multi-scan (**SADABS**; Bruker, 2005 ▶) *T*_min_ = 0.795, *T*_max_ = 0.980
24411 measured reflections
8309 independent reflections
6194 reflections with *I* > 2˘*I*)
*R*_int_ = 0.037

#### Refinement

*R*[*F* ^2^ > 2σ(*F* ^2^)] = 0.053
*wR*(*F* ^2^) = 0.104
*S* = 1.04
8309 reflections
375 parameters
H-atom parameters constrained
Δρ_max_ = 0.50 e Å^−3^
Δρ_min_ = −0.50 e Å^−3^

### 

Data collection: *APEX2* (Bruker, 2005 ▶); cell refinement: *SAINT* (Bruker, 2005 ▶); data reduction: *SAINT*; program(s) used to solve structure: *SHELXTL* (Sheldrick, 2008 ▶); program(s) used to refine structure: *SHELXTL*; molecular graphics: *SHELXTL* software used to prepare material for publication: *SHELXTL* and *PLATON* (Spek, 2009 ▶).

## Supplementary Material

Crystal structure: contains datablocks global, I. DOI: 10.1107/S1600536809014512/lh2805sup1.cif

Structure factors: contains datablocks I. DOI: 10.1107/S1600536809014512/lh2805Isup2.hkl

Additional supplementary materials:  crystallographic information; 3D view; checkCIF report

## Figures and Tables

**Table 1:** Hydrogen-bond geometry (Å, °)

*D*—H⋯*A*	*D*—H	H⋯*A*	*D*⋯*A*	*D*—H⋯*A*
N2*A*—H2*NA*⋯O1*B*^i^	0.81	2.20	3.003 (4)	171
C10*A*—H10*A*⋯O2*A*^ii^	0.95	2.42	3.235 (3)	144
C12*B*—H12*B*⋯O1*A*^ii^	0.95	2.48	3.233 (6)	137
C9*B*—H9*BA*⋯O1*B*^iii^	0.95	2.55	3.369 (9)	145

Symmetry codes: (i) 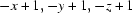; (ii) 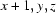; (iii) 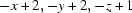.

**Fig. 1.**
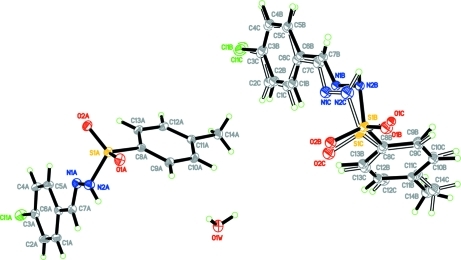
The molecular structure of the title compound, showing 50% probability displacement ellipsoids and the atomic numbering. Open bonds indicate the minor component.

## supplementary crystallographic information

### Comment

Sulfonamides were the first class of antimicrobial agents to be discovered. They inhibit dihydropteroate synthetase in the bacterial folic acid pathway. Although their clinical role has diminished, they are still useful in certain situations because of its efficacy and low cost (Krygowski *et al.*, 1998). Sulfonamides (sulfanilamide, sulfamethoxazole, sulfafurazole) are structural analogues of *p*-aminobenzoic acid (PABA) and compete with PABA to block its conversion to dihydrofolic acid. These agents are generally used in combination with other drugs (usually sulfonamides) to prevent or treat a number of bacterial and parasitic infections (Tierney *et al.*, 2006). Because of the above impotrtant features, we report the crystal structure of the title compound (I).

The title compound, (Fig. I), is a novel sulfonamide derivative. The bond lengths (Allen *et al.*, 1987) and angles are within the normal ranges and are comparable with the related structures (Kia *et al.* 2008*a*,b; Mehrabi *et al.*, 2008; Ali *et al.* 2007). The asymmetric unit of the title compound comprises two crystallographically independent molecules and a water molecule of crystallization which is partially occupied. One of the molecules of the title compound is disordered over two positions with a refined site-occupancy ratio of 0.605 (10)/0.395 (10). Intermolecular N—H···O and C—H···O hydrogen bonds link the neighbouring molecules together (Table 1). The dihedral angles between the two benzene rings in molecules A, B and C are 67.8 (3), 74.6 (5) and 84.96 (11)°, respectively. The crystal structure is further stabilized by intermolecular π-π interactions [*Cg*1···*Cg* 2^iv^ = 3.749 (3) Å, (iv) -1 + *x*, -1 + *y*, *z*; *Cg*2···*Cg*3^v^ = 3.805 (5) Å, (v) 1 + *x*, 1 + *y*, *z*; *Cg*1, *Cg*2 and *Cg*3 are the centroids of the C1B–C6B, C1A–C6A, and C1C–C6C benzene rings].

### Experimental

*p*-Tosylhydrazine (2 mmol) was added to a 50 ml refluxing ethanolic solution of 4-chlorobenzaldehyde (2 mmol). The mixture was stirred for 2 h. After cooling, the colorless crystalline solid was isolated by filtration, washed with cold ethanol, and re-crystallized from ethanol.

### Refinement

The N-bound H atoms were located from the difference Fourier map and constrained to refine with the carrier atom with *U*_iso_(H) = 1.2 *U*_eq_(N). The rest of the hydrogen atoms were positioned geometrically and refined as riding model with *U*_iso_(H) = 1.2 or 1.5 *U*_eq_(C). A rotating group model was used for the methyl groups. For the disordered molecule, only the S and Cl atoms were refined anisotropically. Initially rigid, similarity and simulation restraints were applied. After steady state has been reached, these restraints were removed for the final refinement. There is no restraints used in the final refinement.

### Crystal data

**Table:**

C_14_H_13_ClN_2_O_2_S·0.15H_2_O	*Z* = 4
*M**_r_* = 311.61	*F*(000) = 646
Triclinic, *P*1	*D*_x_ = 1.444 Mg m^−^^3^
Hall symbol: -P 1	Mo *K*α radiation, λ = 0.71073 Å
*a* = 7.9408 (2) Å	Cell parameters from 6610 reflections
*b* = 11.0592 (2) Å	θ = 2.5–31.3°
*c* = 17.7759 (4) Å	µ = 0.42 mm^−^^1^
α = 77.521 (1)°	*T* = 100 K
β = 83.415 (1)°	Needle, colourless
γ = 70.313 (1)°	0.58 × 0.13 × 0.05 mm
*V* = 1433.60 (5) Å^3^	

### Data collection

**Table:**

Bruker SMART APEXII CCD area-detector diffractometer	8309 independent reflections
Radiation source: fine-focus sealed tube	6194 reflections with *I* > 2˘*I*)
graphite	*R*_int_ = 0.037
φ and ω scans	θ_max_ = 30.0°, θ_min_ = 2.0°
Absorption correction: multi-scan (*SADABS*; Bruker, 2005)	*h* = −11→11
*T*_min_ = 0.795, *T*_max_ = 0.980	*k* = −15→15
24411 measured reflections	*l* = −25→24

### Refinement

**Table:**

Refinement on *F*^2^	Primary atom site location: structure-invariant direct methods
Least-squares matrix: full	Secondary atom site location: difference Fourier map
*R*[*F*^2^ > 2σ(*F*^2^)] = 0.053	Hydrogen site location: inferred from neighbouring sites
*wR*(*F*^2^) = 0.104	H-atom parameters constrained
*S* = 1.04	*w* = 1/[σ^2^(*F*_o_^2^) + (0.0215*P*)^2^ + 1.6504*P*] where *P* = (*F*_o_^2^ + 2*F*_c_^2^)/3
8309 reflections	(Δ/σ)_max_ < 0.001
375 parameters	Δρ_max_ = 0.50 e Å^−^^3^
0 restraints	Δρ_min_ = −0.50 e Å^−^^3^

### Special details

**Table:**

Experimental. The crystal was placed in the cold stream of an Oxford Cyrosystems Cobra open-flow nitrogen cryostat (Cosier & Glazer, 1986) operating at 100.0 (1) K.
Geometry. All e.s.d.'s (except the e.s.d. in the dihedral angle between two l.s. planes) are estimated using the full covariance matrix. The cell e.s.d.'s are taken into account individually in the estimation of e.s.d.'s in distances, angles and torsion angles; correlations between e.s.d.'s in cell parameters are only used when they are defined by crystal symmetry. An approximate (isotropic) treatment of cell e.s.d.'s is used for estimating e.s.d.'s involving l.s. planes.
Refinement. Refinement of *F*^2^ against ALL reflections. The weighted *R*-factor *wR* and goodness of fit *S* are based on *F*^2^, conventional *R*-factors *R* are based on *F*, with *F* set to zero for negative *F*^2^. The threshold expression of *F*^2^ > σ(*F*^2^) is used only for calculating *R*-factors(gt) *etc*. and is not relevant to the choice of reflections for refinement. *R*-factors based on *F*^2^ are statistically about twice as large as those based on *F*, and *R*- factors based on ALL data will be even larger.

### Fractional atomic coordinates and isotropic or equivalent isotropic displacement parameters (Å^2^)

**Table:**

	*x*	*y*	*z*	*U*_iso_*/*U*_eq_	Occ. (<1)
Cl1A	−0.03125 (9)	−0.28061 (6)	0.03561 (3)	0.03328 (14)	
S1A	−0.04954 (7)	0.36566 (5)	0.28980 (3)	0.01910 (11)	
O1A	−0.0364 (2)	0.40061 (15)	0.36136 (8)	0.0239 (3)	
O2A	−0.20040 (19)	0.43517 (14)	0.24334 (9)	0.0231 (3)	
N1A	−0.0551 (2)	0.15287 (17)	0.25433 (10)	0.0217 (4)	
N2A	−0.0455 (2)	0.21180 (17)	0.31520 (10)	0.0224 (4)	
H2NA	0.0244	0.1702	0.3488	0.027*	
C1A	0.0749 (3)	−0.1822 (2)	0.22419 (13)	0.0247 (5)	
H1AA	0.1341	−0.2250	0.2708	0.030*	
C2A	0.0692 (3)	−0.2551 (2)	0.17103 (13)	0.0269 (5)	
H2AA	0.1251	−0.3473	0.1805	0.032*	
C3A	−0.0191 (3)	−0.1918 (2)	0.10381 (12)	0.0232 (4)	
C4A	−0.1012 (3)	−0.0572 (2)	0.08878 (12)	0.0245 (5)	
H4AA	−0.1620	−0.0151	0.0424	0.029*	
C5A	−0.0933 (3)	0.0146 (2)	0.14200 (12)	0.0245 (5)	
H5AA	−0.1485	0.1069	0.1320	0.029*	
C6A	−0.0049 (3)	−0.0468 (2)	0.21049 (12)	0.0199 (4)	
C7A	0.0052 (3)	0.0281 (2)	0.26765 (12)	0.0212 (4)	
H7AA	0.0577	−0.0171	0.3155	0.025*	
C8A	0.1444 (3)	0.36812 (19)	0.23169 (11)	0.0176 (4)	
C9A	0.3007 (3)	0.3508 (2)	0.26715 (12)	0.0202 (4)	
H9AA	0.3035	0.3344	0.3218	0.024*	
C10A	0.4523 (3)	0.3578 (2)	0.22155 (12)	0.0213 (4)	
H10A	0.5591	0.3461	0.2455	0.026*	
C11A	0.4509 (3)	0.38171 (19)	0.14111 (12)	0.0198 (4)	
C12A	0.2935 (3)	0.3947 (2)	0.10728 (12)	0.0215 (4)	
H12A	0.2913	0.4081	0.0527	0.026*	
C13A	0.1408 (3)	0.3885 (2)	0.15204 (12)	0.0202 (4)	
H13A	0.0345	0.3981	0.1284	0.024*	
C14A	0.6143 (3)	0.3962 (2)	0.09342 (12)	0.0252 (5)	
H14A	0.7218	0.3418	0.1214	0.038*	
H14B	0.6095	0.4880	0.0834	0.038*	
H14C	0.6182	0.3683	0.0444	0.038*	
Cl1B	0.4426 (5)	0.7959 (3)	0.04028 (16)	0.0210 (7)	0.605 (10)
S1B	0.7628 (6)	0.8594 (5)	0.5026 (3)	0.0168 (5)	0.605 (10)
O1B	0.7828 (5)	0.9112 (4)	0.5594 (2)	0.0267 (11)*	0.605 (10)
O2B	0.6477 (4)	0.7813 (4)	0.52604 (16)	0.0226 (8)*	0.605 (10)
N1B	0.6638 (5)	0.9182 (4)	0.36594 (18)	0.0165 (8)*	0.605 (10)
N2B	0.6954 (5)	0.9726 (3)	0.42438 (17)	0.0156 (8)*	0.605 (10)
H1	0.6290	1.0339	0.4335	0.019*	0.605 (10)
C1B	0.6088 (7)	0.8115 (5)	0.2437 (3)	0.0207 (13)*	0.605 (10)
H1BA	0.6781	0.7524	0.2847	0.025*	0.605 (10)
C2B	0.5833 (9)	0.7648 (7)	0.1792 (4)	0.0226 (17)*	0.605 (10)
H2BA	0.6357	0.6752	0.1757	0.027*	0.605 (10)
C3B	0.4768 (13)	0.8562 (9)	0.1195 (5)	0.023 (3)*	0.605 (10)
C4B	0.3999 (8)	0.9877 (6)	0.1263 (3)	0.0179 (15)*	0.605 (10)
H4BA	0.3282	1.0489	0.0868	0.021*	0.605 (10)
C5B	0.4286 (10)	1.0275 (7)	0.1902 (4)	0.0168 (17)*	0.605 (10)
H5BA	0.3736	1.1166	0.1943	0.020*	0.605 (10)
C6B	0.5323 (8)	0.9447 (6)	0.2476 (3)	0.0161 (14)*	0.605 (10)
C7B	0.5643 (7)	0.9940 (5)	0.3126 (3)	0.0171 (12)*	0.605 (10)
H7BA	0.5099	1.0840	0.3150	0.021*	0.605 (10)
C8B	0.9725 (11)	0.7669 (7)	0.4691 (4)	0.018 (2)*	0.605 (10)
C9B	1.1215 (10)	0.8081 (8)	0.4638 (4)	0.024 (2)*	0.605 (10)
H9BA	1.1045	0.8926	0.4737	0.029*	0.605 (10)
C10B	1.2837 (10)	0.7368 (8)	0.4460 (4)	0.0206 (19)*	0.605 (10)
H10B	1.3840	0.7627	0.4508	0.025*	0.605 (10)
C11B	1.3073 (8)	0.6155 (6)	0.4185 (3)	0.0176 (14)*	0.605 (10)
C12B	1.1562 (8)	0.5784 (5)	0.4180 (3)	0.0178 (13)*	0.605 (10)
H12B	1.1679	0.5008	0.4002	0.021*	0.605 (10)
C13B	0.9882 (8)	0.6530 (5)	0.4434 (3)	0.0171 (13)*	0.605 (10)
H13B	0.8862	0.6267	0.4431	0.021*	0.605 (10)
C14B	1.4892 (8)	0.5349 (6)	0.3909 (3)	0.0273 (14)*	0.605 (10)
H14D	1.4993	0.4421	0.4037	0.041*	0.605 (10)
H14E	1.5824	0.5492	0.4162	0.041*	0.605 (10)
H14F	1.5039	0.5613	0.3349	0.041*	0.605 (10)
Cl1C	0.4463 (11)	0.7958 (8)	0.0393 (4)	0.051 (2)	0.395 (10)
S1C	0.7706 (11)	0.8308 (8)	0.5037 (5)	0.0256 (13)	0.395 (10)
O1C	0.7853 (7)	0.9379 (6)	0.5554 (3)	0.0141 (12)*	0.395 (10)
O2C	0.6787 (7)	0.7358 (7)	0.5268 (3)	0.0280 (13)*	0.395 (10)
N1C	0.6343 (8)	0.8860 (7)	0.3737 (3)	0.0255 (14)*	0.395 (10)
N2C	0.6538 (9)	0.9402 (7)	0.4344 (3)	0.0313 (15)*	0.395 (10)
H2	0.6285	1.0343	0.4335	0.038*	0.395 (10)
C1C	0.5969 (12)	0.7927 (8)	0.2403 (5)	0.022 (2)*	0.395 (10)
H1CA	0.6645	0.7295	0.2803	0.027*	0.395 (10)
C2C	0.5644 (14)	0.7541 (11)	0.1793 (6)	0.025 (3)*	0.395 (10)
H2CA	0.6037	0.6637	0.1770	0.030*	0.395 (10)
C3C	0.4761 (19)	0.8428 (13)	0.1213 (8)	0.019 (4)*	0.395 (10)
C4C	0.4100 (13)	0.9730 (9)	0.1186 (5)	0.020 (3)*	0.395 (10)
H4CA	0.3468	1.0308	0.0757	0.024*	0.395 (10)
C5C	0.4388 (17)	1.0197 (13)	0.1822 (7)	0.028 (4)*	0.395 (10)
H5CA	0.3978	1.1105	0.1833	0.033*	0.395 (10)
C6C	0.5347 (13)	0.9227 (9)	0.2467 (5)	0.021 (2)*	0.395 (10)
C7C	0.5617 (12)	0.9691 (9)	0.3144 (5)	0.025 (2)*	0.395 (10)
H7CA	0.5256	1.0602	0.3143	0.030*	0.395 (10)
C8C	0.9811 (16)	0.7516 (11)	0.4672 (7)	0.017 (3)*	0.395 (10)
C9C	1.1153 (13)	0.8047 (10)	0.4739 (6)	0.013 (2)*	0.395 (10)
H9CA	1.0968	0.8759	0.4992	0.016*	0.395 (10)
C10C	1.2924 (17)	0.7361 (12)	0.4364 (6)	0.023 (3)*	0.395 (10)
H10C	1.3829	0.7765	0.4288	0.028*	0.395 (10)
C11C	1.3283 (12)	0.6282 (9)	0.4145 (5)	0.021 (2)*	0.395 (10)
C12C	1.1917 (13)	0.5762 (10)	0.4146 (5)	0.030 (3)*	0.395 (10)
H12C	1.2186	0.4964	0.3968	0.036*	0.395 (10)
C13C	1.0183 (13)	0.6363 (9)	0.4394 (5)	0.024 (2)*	0.395 (10)
H13C	0.9264	0.6000	0.4376	0.029*	0.395 (10)
C14C	1.5123 (12)	0.5534 (9)	0.3901 (5)	0.032 (2)*	0.395 (10)
H14G	1.5942	0.6014	0.3931	0.047*	0.395 (10)
H14H	1.5133	0.5418	0.3369	0.047*	0.395 (10)
H14I	1.5509	0.4675	0.4242	0.047*	0.395 (10)
O1W	0.6383 (7)	0.1913 (5)	0.3920 (3)	0.0265 (11)	0.30
H1W1	0.5425	0.2376	0.4121	0.040*	0.30
H2W1	0.6993	0.2411	0.3717	0.040*	0.30

### Atomic displacement parameters (Å^2^)

**Table:**

	*U*^11^	*U*^22^	*U*^33^	*U*^12^	*U*^13^	*U*^23^
Cl1A	0.0387 (3)	0.0291 (3)	0.0325 (3)	−0.0048 (3)	−0.0059 (3)	−0.0144 (2)
S1A	0.0180 (2)	0.0190 (2)	0.0211 (2)	−0.0045 (2)	−0.00232 (19)	−0.00677 (19)
O1A	0.0240 (8)	0.0278 (8)	0.0226 (7)	−0.0083 (7)	−0.0004 (6)	−0.0112 (6)
O2A	0.0178 (7)	0.0228 (8)	0.0283 (8)	−0.0039 (6)	−0.0045 (6)	−0.0060 (6)
N1A	0.0239 (9)	0.0222 (9)	0.0209 (8)	−0.0090 (8)	−0.0017 (7)	−0.0054 (7)
N2A	0.0275 (10)	0.0220 (9)	0.0185 (8)	−0.0080 (8)	−0.0054 (7)	−0.0033 (7)
C1A	0.0266 (11)	0.0213 (11)	0.0246 (11)	−0.0063 (9)	−0.0071 (9)	0.0000 (8)
C2A	0.0308 (12)	0.0166 (10)	0.0314 (12)	−0.0035 (9)	−0.0071 (10)	−0.0042 (9)
C3A	0.0233 (11)	0.0229 (11)	0.0252 (10)	−0.0071 (9)	−0.0007 (9)	−0.0089 (9)
C4A	0.0243 (11)	0.0229 (11)	0.0231 (10)	−0.0030 (9)	−0.0062 (9)	−0.0025 (8)
C5A	0.0254 (11)	0.0177 (10)	0.0273 (11)	−0.0024 (9)	−0.0058 (9)	−0.0029 (8)
C6A	0.0204 (10)	0.0199 (10)	0.0203 (10)	−0.0088 (8)	0.0005 (8)	−0.0030 (8)
C7A	0.0208 (10)	0.0223 (10)	0.0210 (10)	−0.0079 (9)	−0.0038 (8)	−0.0022 (8)
C8A	0.0168 (9)	0.0132 (9)	0.0219 (9)	−0.0026 (8)	−0.0018 (8)	−0.0050 (7)
C9A	0.0213 (10)	0.0211 (10)	0.0189 (9)	−0.0055 (8)	−0.0040 (8)	−0.0055 (8)
C10A	0.0184 (10)	0.0235 (10)	0.0235 (10)	−0.0061 (9)	−0.0056 (8)	−0.0060 (8)
C11A	0.0198 (10)	0.0152 (9)	0.0240 (10)	−0.0039 (8)	−0.0017 (8)	−0.0054 (8)
C12A	0.0222 (11)	0.0212 (10)	0.0190 (10)	−0.0036 (9)	−0.0040 (8)	−0.0036 (8)
C13A	0.0187 (10)	0.0191 (10)	0.0221 (10)	−0.0029 (8)	−0.0062 (8)	−0.0050 (8)
C14A	0.0231 (11)	0.0280 (11)	0.0248 (11)	−0.0087 (9)	−0.0004 (9)	−0.0052 (9)
Cl1B	0.0247 (16)	0.0205 (15)	0.0174 (12)	−0.0042 (13)	−0.0037 (11)	−0.0063 (10)
S1B	0.0124 (7)	0.0205 (16)	0.0172 (6)	−0.0047 (9)	−0.0004 (5)	−0.0040 (9)
Cl1C	0.049 (4)	0.046 (4)	0.057 (4)	−0.006 (3)	−0.019 (3)	−0.013 (3)
S1C	0.0256 (14)	0.027 (3)	0.0219 (12)	−0.0022 (17)	−0.0113 (9)	−0.0058 (18)
O1W	0.024 (3)	0.031 (3)	0.027 (3)	−0.011 (2)	0.001 (2)	−0.009 (2)

### Geometric parameters (Å, °)

**Table:**

Cl1A—C3A	1.746 (2)	C6B—C7B	1.459 (7)
S1A—O2A	1.4284 (15)	C7B—H7BA	0.9500
S1A—O1A	1.4314 (14)	C8B—C9B	1.392 (11)
S1A—N2A	1.6538 (18)	C8B—C13B	1.393 (9)
S1A—C8A	1.758 (2)	C9B—C10B	1.308 (11)
N1A—C7A	1.277 (3)	C9B—H9BA	0.9500
N1A—N2A	1.399 (2)	C10B—C11B	1.473 (10)
N2A—H2NA	0.8135	C10B—H10B	0.9500
C1A—C2A	1.382 (3)	C11B—C12B	1.394 (8)
C1A—C6A	1.393 (3)	C11B—C14B	1.510 (8)
C1A—H1AA	0.9500	C12B—C13B	1.395 (7)
C2A—C3A	1.382 (3)	C12B—H12B	0.9500
C2A—H2AA	0.9500	C13B—H13B	0.9500
C3A—C4A	1.387 (3)	C14B—H14D	0.9800
C4A—C5A	1.379 (3)	C14B—H14E	0.9800
C4A—H4AA	0.9500	C14B—H14F	0.9800
C5A—C6A	1.398 (3)	Cl1C—C3C	1.714 (15)
C5A—H5AA	0.9500	S1C—O2C	1.438 (9)
C6A—C7A	1.467 (3)	S1C—N2C	1.644 (10)
C7A—H7AA	0.9500	S1C—O1C	1.687 (11)
C8A—C13A	1.387 (3)	S1C—C8C	1.729 (14)
C8A—C9A	1.394 (3)	N1C—C7C	1.286 (10)
C9A—C10A	1.387 (3)	N1C—N2C	1.389 (8)
C9A—H9AA	0.9500	N2C—H1	0.9839
C10A—C11A	1.397 (3)	N2C—H2	0.9876
C10A—H10A	0.9500	C1C—C2C	1.327 (13)
C11A—C12A	1.400 (3)	C1C—C6C	1.380 (12)
C11A—C14A	1.502 (3)	C1C—H1CA	0.9500
C12A—C13A	1.385 (3)	C2C—C3C	1.335 (17)
C12A—H12A	0.9500	C2C—H2CA	0.9500
C13A—H13A	0.9500	C3C—C4C	1.349 (16)
C14A—H14A	0.9800	C4C—C5C	1.410 (14)
C14A—H14B	0.9800	C4C—H4CA	0.9500
C14A—H14C	0.9800	C5C—C6C	1.471 (15)
Cl1B—C3B	1.765 (10)	C5C—H5CA	0.9500
S1B—O1B	1.312 (7)	C6C—C7C	1.468 (12)
S1B—O2B	1.430 (5)	C7C—H7CA	0.9500
S1B—N2B	1.660 (6)	C8C—C13C	1.394 (14)
S1B—C8B	1.750 (9)	C8C—C9C	1.405 (15)
N1B—C7B	1.268 (6)	C9C—C10C	1.511 (16)
N1B—N2B	1.391 (4)	C9C—H9CA	0.9500
N2B—H1	0.7419	C10C—C11C	1.266 (15)
N2B—H2	0.7467	C10C—H10C	0.9500
C1B—C6B	1.406 (7)	C11C—C12C	1.388 (13)
C1B—C2B	1.416 (8)	C11C—C14C	1.485 (13)
C1B—H1BA	0.9500	C12C—C13C	1.380 (12)
C2B—C3B	1.424 (11)	C12C—H12C	0.9500
C2B—H2BA	0.9500	C13C—H13C	0.9500
C3B—C4B	1.401 (11)	C14C—H14G	0.9800
C4B—C5B	1.369 (9)	C14C—H14H	0.9800
C4B—H4BA	0.9500	C14C—H14I	0.9800
C5B—C6B	1.357 (9)	O1W—H1W1	0.8500
C5B—H5BA	0.9500	O1W—H2W1	0.8500
			
O2A—S1A—O1A	120.90 (9)	C5B—C6B—C1B	119.4 (5)
O2A—S1A—N2A	107.50 (9)	C5B—C6B—C7B	120.1 (5)
O1A—S1A—N2A	103.58 (9)	C1B—C6B—C7B	120.5 (5)
O2A—S1A—C8A	107.70 (9)	N1B—C7B—C6B	120.8 (5)
O1A—S1A—C8A	108.72 (9)	N1B—C7B—H7BA	119.6
N2A—S1A—C8A	107.78 (9)	C6B—C7B—H7BA	119.6
C7A—N1A—N2A	115.35 (17)	C9B—C8B—C13B	119.3 (7)
N1A—N2A—S1A	115.08 (13)	C9B—C8B—S1B	121.4 (6)
N1A—N2A—H2NA	118.9	C13B—C8B—S1B	119.1 (6)
S1A—N2A—H2NA	112.4	C10B—C9B—C8B	123.5 (8)
C2A—C1A—C6A	121.1 (2)	C10B—C9B—H9BA	118.2
C2A—C1A—H1AA	119.5	C8B—C9B—H9BA	118.2
C6A—C1A—H1AA	119.5	C9B—C10B—C11B	118.5 (7)
C1A—C2A—C3A	118.9 (2)	C9B—C10B—H10B	120.8
C1A—C2A—H2AA	120.6	C11B—C10B—H10B	120.8
C3A—C2A—H2AA	120.6	C12B—C11B—C10B	117.9 (5)
C2A—C3A—C4A	121.4 (2)	C12B—C11B—C14B	121.3 (5)
C2A—C3A—Cl1A	120.30 (17)	C10B—C11B—C14B	120.9 (5)
C4A—C3A—Cl1A	118.27 (16)	C11B—C12B—C13B	121.2 (5)
C5A—C4A—C3A	119.1 (2)	C11B—C12B—H12B	119.4
C5A—C4A—H4AA	120.4	C13B—C12B—H12B	119.4
C3A—C4A—H4AA	120.4	C8B—C13B—C12B	118.9 (5)
C4A—C5A—C6A	120.7 (2)	C8B—C13B—H13B	120.5
C4A—C5A—H5AA	119.7	C12B—C13B—H13B	120.5
C6A—C5A—H5AA	119.7	O2C—S1C—N2C	105.3 (6)
C1A—C6A—C5A	118.80 (19)	O2C—S1C—O1C	127.0 (6)
C1A—C6A—C7A	119.75 (18)	N2C—S1C—O1C	96.6 (5)
C5A—C6A—C7A	121.45 (19)	O2C—S1C—C8C	107.3 (6)
N1A—C7A—C6A	121.20 (19)	N2C—S1C—C8C	109.7 (6)
N1A—C7A—H7AA	119.4	O1C—S1C—C8C	109.6 (6)
C6A—C7A—H7AA	119.4	C7C—N1C—N2C	115.1 (6)
C13A—C8A—C9A	120.8 (2)	N1C—N2C—S1C	112.8 (5)
C13A—C8A—S1A	120.46 (16)	N1C—N2C—H1	125.9
C9A—C8A—S1A	118.74 (15)	S1C—N2C—H1	119.7
C10A—C9A—C8A	119.04 (18)	N1C—N2C—H2	125.9
C10A—C9A—H9AA	120.5	S1C—N2C—H2	119.8
C8A—C9A—H9AA	120.5	H1—N2C—H2	0.2
C9A—C10A—C11A	121.31 (19)	C2C—C1C—C6C	121.4 (9)
C9A—C10A—H10A	119.3	C2C—C1C—H1CA	119.3
C11A—C10A—H10A	119.3	C6C—C1C—H1CA	119.3
C10A—C11A—C12A	118.3 (2)	C1C—C2C—C3C	119.7 (11)
C10A—C11A—C14A	119.89 (19)	C1C—C2C—H2CA	120.2
C12A—C11A—C14A	121.82 (19)	C3C—C2C—H2CA	120.2
C13A—C12A—C11A	121.07 (19)	C2C—C3C—C4C	125.8 (12)
C13A—C12A—H12A	119.5	C2C—C3C—Cl1C	120.3 (10)
C11A—C12A—H12A	119.5	C4C—C3C—Cl1C	113.9 (10)
C12A—C13A—C8A	119.46 (19)	C3C—C4C—C5C	117.1 (10)
C12A—C13A—H13A	120.3	C3C—C4C—H4CA	121.5
C8A—C13A—H13A	120.3	C5C—C4C—H4CA	121.5
C11A—C14A—H14A	109.5	C4C—C5C—C6C	117.7 (10)
C11A—C14A—H14B	109.5	C4C—C5C—H5CA	121.2
H14A—C14A—H14B	109.5	C6C—C5C—H5CA	121.2
C11A—C14A—H14C	109.5	C1C—C6C—C7C	123.2 (8)
H14A—C14A—H14C	109.5	C1C—C6C—C5C	118.3 (8)
H14B—C14A—H14C	109.5	C7C—C6C—C5C	118.5 (8)
O1B—S1B—O2B	111.1 (4)	N1C—C7C—C6C	119.8 (8)
O1B—S1B—N2B	111.4 (4)	N1C—C7C—H7CA	120.1
O2B—S1B—N2B	112.5 (4)	C6C—C7C—H7CA	120.1
O1B—S1B—C8B	109.7 (4)	C13C—C8C—C9C	122.2 (10)
O2B—S1B—C8B	111.0 (4)	C13C—C8C—S1C	122.1 (9)
N2B—S1B—C8B	100.7 (4)	C9C—C8C—S1C	115.5 (8)
C7B—N1B—N2B	117.9 (4)	C8C—C9C—C10C	112.5 (10)
N1B—N2B—S1B	111.1 (3)	C8C—C9C—H9CA	123.7
N1B—N2B—H1	119.5	C10C—C9C—H9CA	123.7
S1B—N2B—H1	112.7	C11C—C10C—C9C	124.3 (11)
N1B—N2B—H2	119.5	C11C—C10C—H10C	117.8
S1B—N2B—H2	112.7	C9C—C10C—H10C	117.8
H1—N2B—H2	0.0	C10C—C11C—C12C	119.1 (10)
C6B—C1B—C2B	120.3 (5)	C10C—C11C—C14C	122.4 (9)
C6B—C1B—H1BA	119.8	C12C—C11C—C14C	118.5 (8)
C2B—C1B—H1BA	119.8	C13C—C12C—C11C	122.4 (9)
C1B—C2B—C3B	118.1 (6)	C13C—C12C—H12C	118.8
C1B—C2B—H2BA	121.0	C11C—C12C—H12C	118.8
C3B—C2B—H2BA	121.0	C12C—C13C—C8C	118.4 (9)
C4B—C3B—C2B	119.8 (7)	C12C—C13C—H13C	120.8
C4B—C3B—Cl1B	122.7 (7)	C8C—C13C—H13C	120.8
C2B—C3B—Cl1B	117.5 (6)	C11C—C14C—H14G	109.5
C5B—C4B—C3B	119.8 (6)	C11C—C14C—H14H	109.5
C5B—C4B—H4BA	120.1	H14G—C14C—H14H	109.5
C3B—C4B—H4BA	120.1	C11C—C14C—H14I	109.5
C6B—C5B—C4B	122.6 (6)	H14G—C14C—H14I	109.5
C6B—C5B—H5BA	118.7	H14H—C14C—H14I	109.5
C4B—C5B—H5BA	118.7	H1W1—O1W—H2W1	107.7
			
C7A—N1A—N2A—S1A	157.97 (16)	O1B—S1B—C8B—C9B	−38.3 (8)
O2A—S1A—N2A—N1A	51.52 (17)	O2B—S1B—C8B—C9B	−161.5 (6)
O1A—S1A—N2A—N1A	−179.42 (15)	N2B—S1B—C8B—C9B	79.2 (7)
C8A—S1A—N2A—N1A	−64.33 (17)	O1B—S1B—C8B—C13B	145.1 (6)
C6A—C1A—C2A—C3A	−0.7 (4)	O2B—S1B—C8B—C13B	21.9 (8)
C1A—C2A—C3A—C4A	0.2 (4)	N2B—S1B—C8B—C13B	−97.4 (6)
C1A—C2A—C3A—Cl1A	−179.37 (18)	C13B—C8B—C9B—C10B	−9.9 (11)
C2A—C3A—C4A—C5A	0.4 (4)	S1B—C8B—C9B—C10B	173.4 (6)
Cl1A—C3A—C4A—C5A	179.92 (18)	C8B—C9B—C10B—C11B	9.7 (11)
C3A—C4A—C5A—C6A	−0.4 (3)	C9B—C10B—C11B—C12B	−4.4 (9)
C2A—C1A—C6A—C5A	0.7 (3)	C9B—C10B—C11B—C14B	174.9 (6)
C2A—C1A—C6A—C7A	−179.3 (2)	C10B—C11B—C12B—C13B	−0.5 (8)
C4A—C5A—C6A—C1A	−0.2 (3)	C14B—C11B—C12B—C13B	−179.8 (5)
C4A—C5A—C6A—C7A	179.8 (2)	C9B—C8B—C13B—C12B	4.5 (9)
N2A—N1A—C7A—C6A	176.81 (18)	S1B—C8B—C13B—C12B	−178.8 (5)
C1A—C6A—C7A—N1A	174.9 (2)	C11B—C12B—C13B—C8B	0.3 (8)
C5A—C6A—C7A—N1A	−5.1 (3)	C7C—N1C—N2C—S1C	−172.5 (7)
O2A—S1A—C8A—C13A	−22.24 (19)	O2C—S1C—N2C—N1C	−56.5 (7)
O1A—S1A—C8A—C13A	−154.88 (16)	O1C—S1C—N2C—N1C	172.3 (5)
N2A—S1A—C8A—C13A	93.47 (17)	C8C—S1C—N2C—N1C	58.8 (8)
O2A—S1A—C8A—C9A	157.01 (15)	C6C—C1C—C2C—C3C	2.8 (16)
O1A—S1A—C8A—C9A	24.38 (19)	C1C—C2C—C3C—C4C	−2(2)
N2A—S1A—C8A—C9A	−87.28 (17)	C1C—C2C—C3C—Cl1C	175.5 (9)
C13A—C8A—C9A—C10A	1.8 (3)	C2C—C3C—C4C—C5C	0.9 (19)
S1A—C8A—C9A—C10A	−177.46 (16)	Cl1C—C3C—C4C—C5C	−176.4 (9)
C8A—C9A—C10A—C11A	−0.1 (3)	C3C—C4C—C5C—C6C	−1.3 (16)
C9A—C10A—C11A—C12A	−1.9 (3)	C2C—C1C—C6C—C7C	177.6 (9)
C9A—C10A—C11A—C14A	176.56 (20)	C2C—C1C—C6C—C5C	−3.3 (14)
C10A—C11A—C12A—C13A	2.1 (3)	C4C—C5C—C6C—C1C	2.5 (15)
C14A—C11A—C12A—C13A	−176.3 (2)	C4C—C5C—C6C—C7C	−178.3 (9)
C11A—C12A—C13A—C8A	−0.5 (3)	N2C—N1C—C7C—C6C	179.9 (7)
C9A—C8A—C13A—C12A	−1.5 (3)	C1C—C6C—C7C—N1C	−5.5 (14)
S1A—C8A—C13A—C12A	177.69 (16)	C5C—C6C—C7C—N1C	175.4 (9)
C7B—N1B—N2B—S1B	159.7 (4)	O2C—S1C—C8C—C13C	22.1 (12)
O1B—S1B—N2B—N1B	−176.3 (3)	N2C—S1C—C8C—C13C	−91.8 (10)
O2B—S1B—N2B—N1B	−50.7 (4)	O1C—S1C—C8C—C13C	163.3 (9)
C8B—S1B—N2B—N1B	67.5 (4)	O2C—S1C—C8C—C9C	−152.4 (8)
C6B—C1B—C2B—C3B	−0.8 (9)	N2C—S1C—C8C—C9C	93.7 (10)
C1B—C2B—C3B—C4B	−0.5 (11)	O1C—S1C—C8C—C9C	−11.2 (11)
C1B—C2B—C3B—Cl1B	−178.8 (5)	C13C—C8C—C9C—C10C	9.1 (15)
C2B—C3B—C4B—C5B	0.4 (12)	S1C—C8C—C9C—C10C	−176.4 (8)
Cl1B—C3B—C4B—C5B	178.5 (6)	C8C—C9C—C10C—C11C	−12.8 (15)
C3B—C4B—C5B—C6B	1.2 (11)	C9C—C10C—C11C—C12C	9.3 (16)
C4B—C5B—C6B—C1B	−2.5 (10)	C9C—C10C—C11C—C14C	−169.5 (9)
C4B—C5B—C6B—C7B	177.3 (6)	C10C—C11C—C12C—C13C	−1.6 (15)
C2B—C1B—C6B—C5B	2.2 (9)	C14C—C11C—C12C—C13C	177.2 (9)
C2B—C1B—C6B—C7B	−177.5 (5)	C11C—C12C—C13C—C8C	−1.7 (14)
N2B—N1B—C7B—C6B	177.1 (4)	C9C—C8C—C13C—C12C	−2.9 (15)
C5B—C6B—C7B—N1B	−178.6 (6)	S1C—C8C—C13C—C12C	−177.1 (8)
C1B—C6B—C7B—N1B	1.2 (8)		

### Hydrogen-bond geometry (Å, °)

**Table:**

*D*—H···*A*	*D*—H	H···*A*	*D*···*A*	*D*—H···*A*
N2A—H2NA···O1B^i^	0.81	2.20	3.003 (4)	171
C10A—H10A···O2A^ii^	0.95	2.42	3.235 (3)	144
C12B—H12B···O1A^ii^	0.95	2.48	3.233 (6)	137
C9B—H9BA···O1B^iii^	0.95	2.55	3.369 (9)	145

Symmetry codes: (i) −*x*+1, −*y*+1, −*z*+1; (ii) *x*+1, *y*, *z*; (iii) −*x*+2, −*y*+2, −*z*+1.
